# Supplementary material for: A clinical transcriptome approach to patient stratification and therapy selection in acute myeloid leukemia
Source: Nat Commun. 2021 Apr 30;12:2474. doi: 10.1038/s41467-021-22625-y (PMC8087683; doi:10.1038/s41467-021-22625-y)
Supplement: Supplementary file 1 — Supplementary Information [file 41467_2021_22625_MOESM1_ESM.pdf]

Supplementary Information

Supplementary Figures

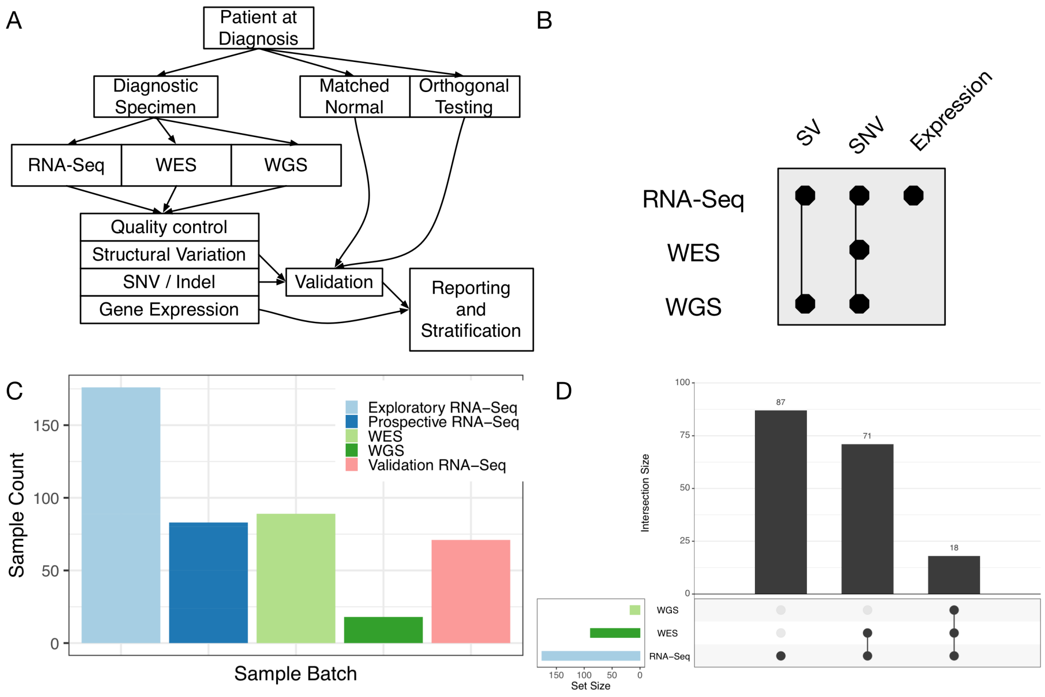

Supplementary Figure 1. Experimental design overview. (**A**) Experimental workflow for the exploratory RNA-Seq, WES, and WGS libraries. Diagnostic tumour specimens were obtained for each patient, with matched normal material used to validate selected somatic SNV and short indel alterations. (**B**) Informatic analyses performed for each sequencing platform. SV: structural variation, SNV: single nucleotide variant. (**C**) Sample count by project batch and sequencing platform. (**D**) Patient samples with matched RNA-Seq, WES, and WGS libraries in the exploratory cohort.

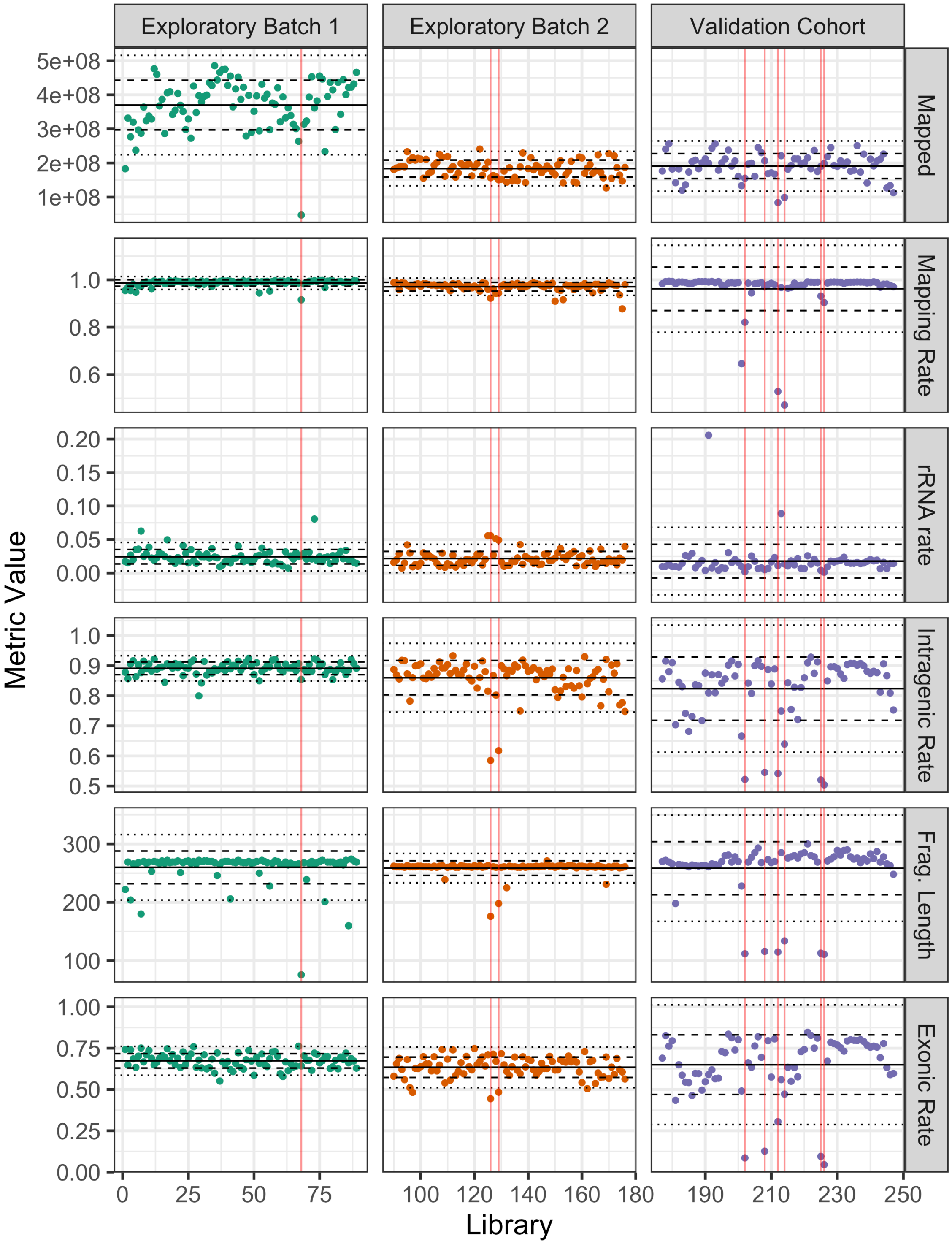
Exploratory Batch 1
Exploratory Batch 2
Validation Cohort
Mapped
Mapping Rate
rRNA rate
Intragenic Rate
Frag. Length
Exonic Rate
Metric Value
Library
5e+08
4e+08
3e+08
2e+08
1e+08
1.0
0.8
0.6
0.20
0.15
0.10
0.05
0.00
1.0
0.9
0.8
0.7
0.6
0.5
300
200
100
1.00
0.75
0.50
0.25
0.00
0
25
50
75
100
120
140
160
180
190
210
230
250

Supplementary Figure 2. Levey-Jennings quality control charts for selected quality metrics for RNA-Seq libraries from the AML PMP. In each panel, the x-axis represents the sample number within the project, while y-axis represents a distinct quality metric. The solid horizontal line represents the mean value for each metric within each sequencing batch, while the dashed and dotted lines represent one and two standard deviations away from the mean, respectively. 'Mapped': total number of mapped reads. 'Mapping Rate': proportion of total reads which were mapped to the reference genome. 'rRNA rate': proportion of reads originating from rRNAs. 'Intragenic rate': proportion of reads that map within genes. 'Fragment length': mean observed fragment length. 'Exonic rate': proportion of reads mapping within exons. Failed samples are indicated with red vertical lines.

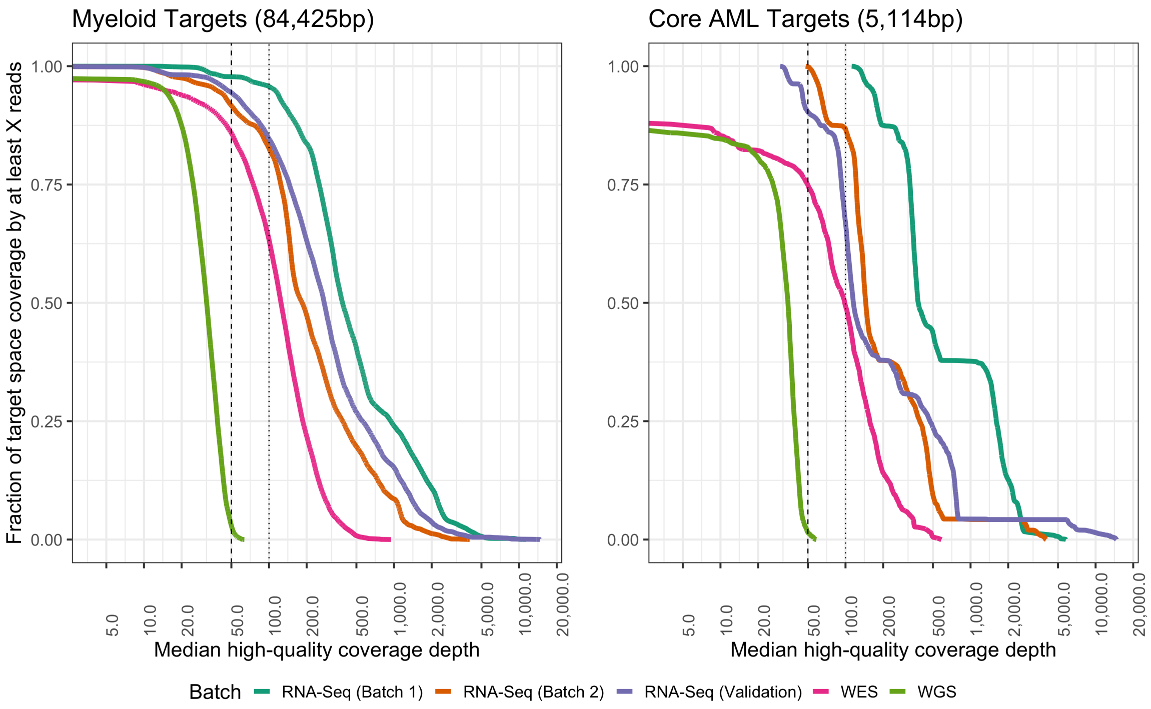

Supplementary Figure 3. Empirical distribution for sequence coverage depth. Myeloid panel targets (left panel) and core clinical targets (*CEBPA*, *DNMT3A*, *FLT3*, *IDH1*, *IDH2*, *KIT*, and *NPM1*) (right panel). The y-axis represents the proportion of bases covered by at least the number of reads indicated by the x-axis. Vertical dashed lines indicate coverage thresholds of 50x and 100x. Sequencing platform and study batch are indicated by line colour.

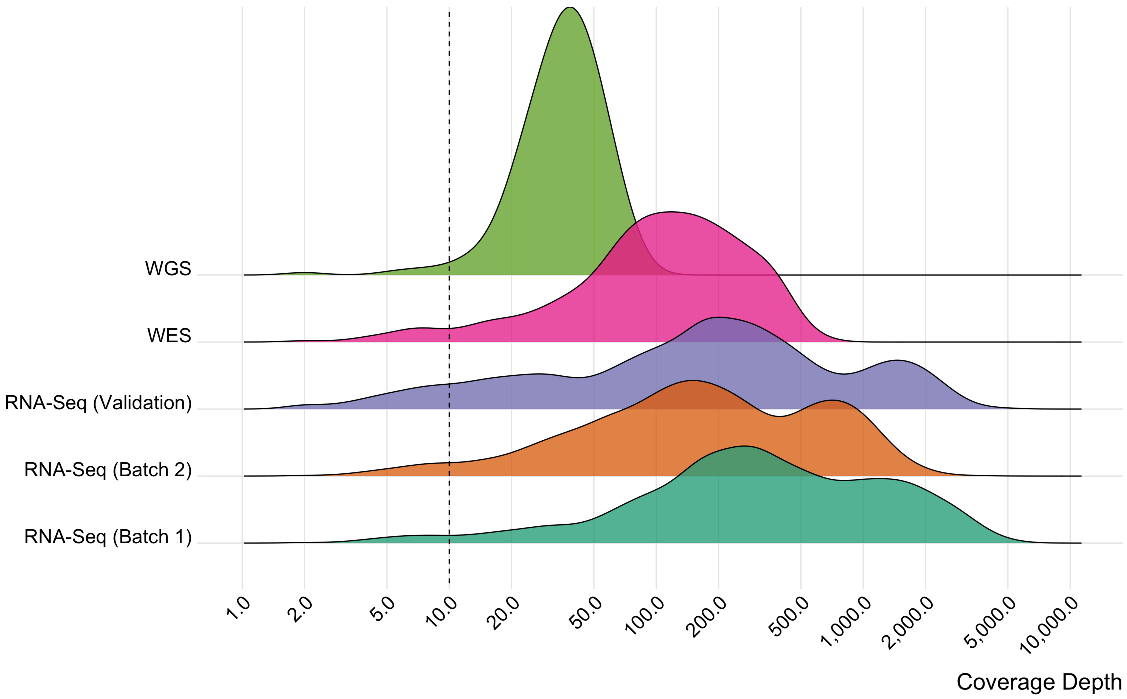

Supplementary Figure 4. Observed sequencing coverage depth for called variants across sequencing platforms and batches in the AML PMP cohort. Sample groups are coloured as in Supplementary Figure 3.

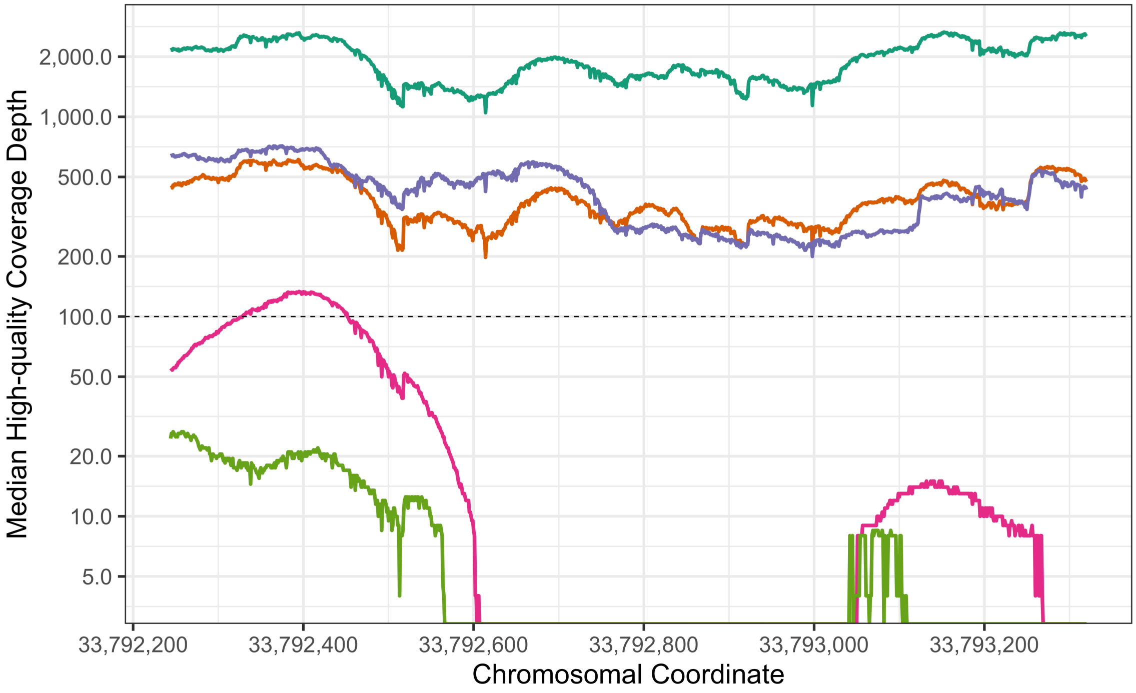

Supplementary Figure 5. Median high-quality sequence coverage depth over *CEBPA* in the AML PMP cohort. Each point represents the median coverage across all samples for a particular sequencing platform and batch. The 'Chromosomal Coordinate' indicates the hg19 position along chromosome 19. The line colour indicates the sequencing platform and study batch, as in Supplementary Figure 3.

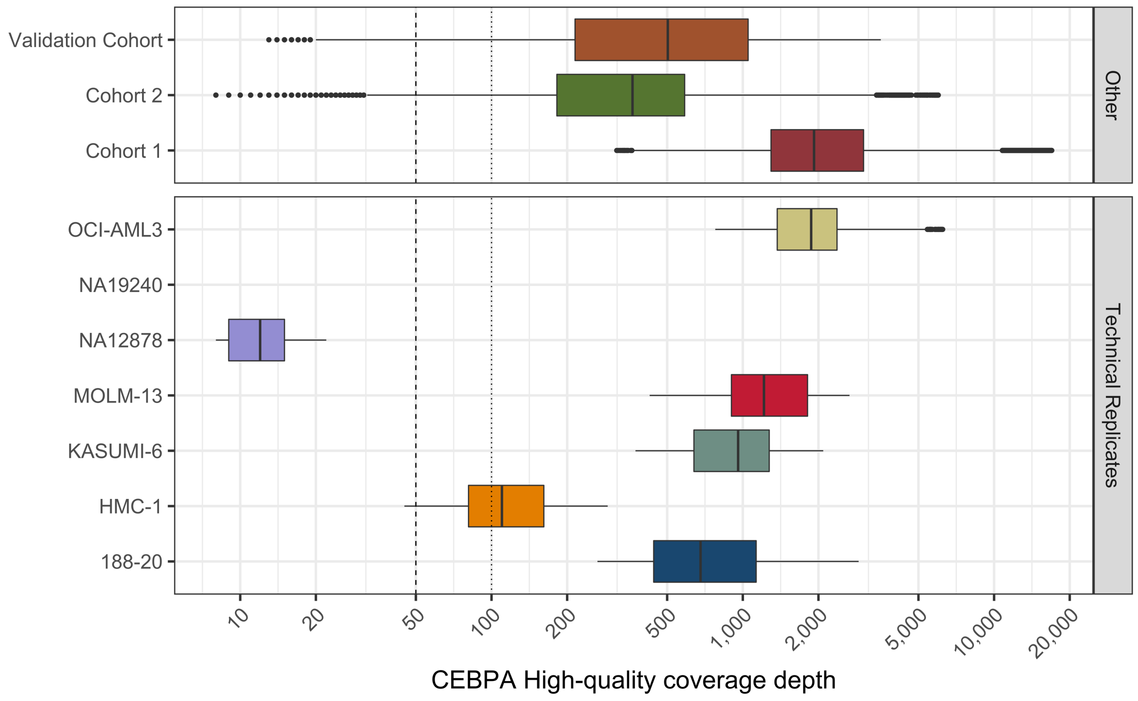

Supplementary Figure 6. High-quality coverage depth in *CEBPA* for technical replicate samples compared to other RNA-Seq and ssRNA-Seq samples. For each sample, high-quality sequence coverage depth at 1,077 sites in *CEBPA* were assessed. Each box plot represents pooled data across retained samples from Cohort 1 (n = 88), Cohort 2 (n = 85), and the RNA-Seq Validation Cohort (n = 28). For cell lines OCI-AML3, NA19240, NA12878, MOLM-13, KASUMI-6, HMC-1, and patient sample 188-20, three technical replicates per condition are shown. For all box plots, the median is shown with a vertical line, with the edges of the box corresponding to the first and third quartiles of the distribution, with whiskers extending to 1.5 * the inter-quartile range of the distribution.

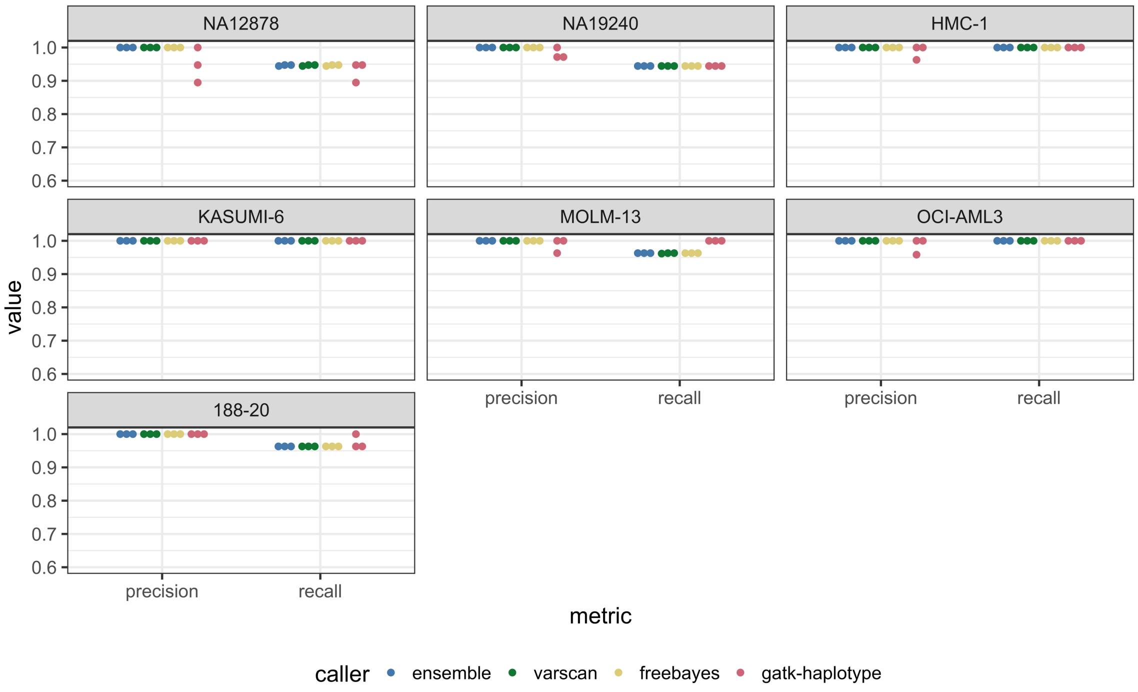

Supplementary Figure 7. Precision and recall by sample and variant caller for replicated RNA-Seq libraries from the AML PMP validation cohort. Precision: TP / (TP + FP), Recall: TP / (TP + FN). Libraries which failed sample QC were excluded.

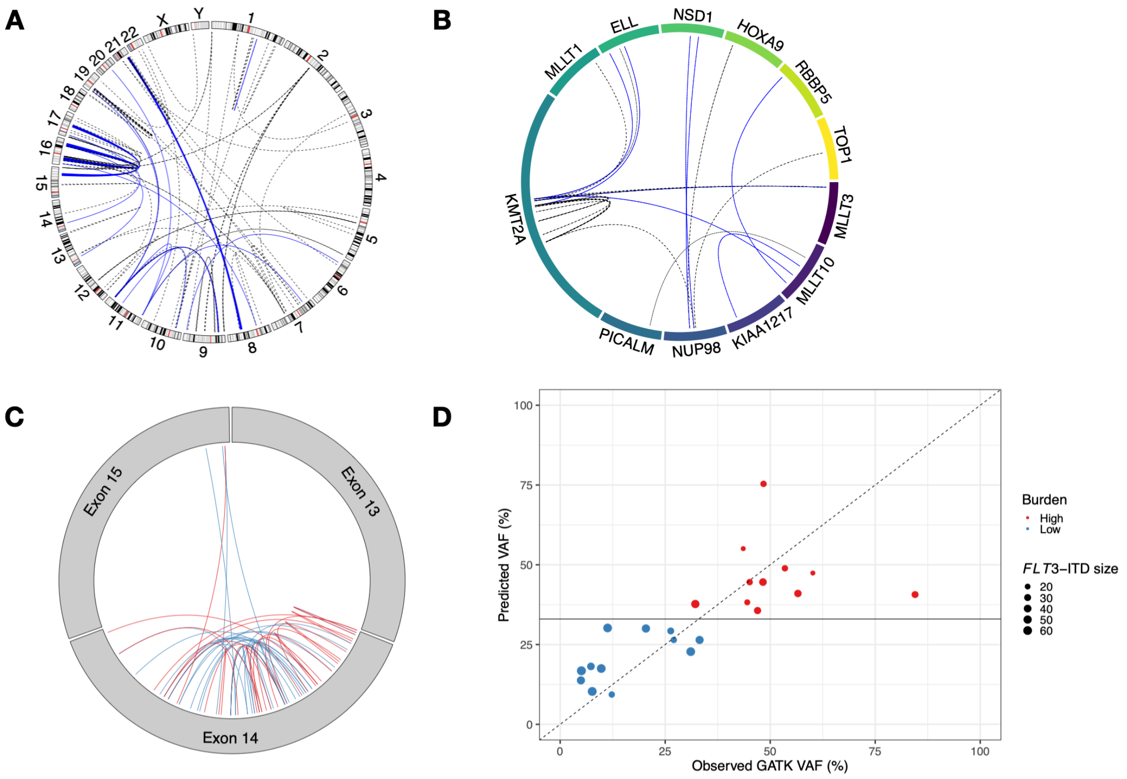

Supplementary Figure 8. RNA-Seq structural variation in the AML PMP exploratory cohorts. (**A**) Filtered gene fusion events detected by RNA-Seq. Each arc represents a distinct set of fusion partners, for known (blue) and novel (black) rearrangements. (**B**) Structural rearrangements involving *KMT2A*-related genes. Each gene (except *KMT2A*) is scaled to the same size, for known (blue) and novel (black) events. (**C**) Intra-gene structural variation in *FLT3*. Each circle segment represents a single exon of *FLT3*, and the zoomed panels show the coordinates of internal tandem duplication breakpoints. (**D**) Predicted VAF for *FLT3*-ITD events based on linear modelling, compared to the observed VAF from GATK HaplotypeCaller for *FLT3*-ITD events detected by both GATK and trans-ABySS.

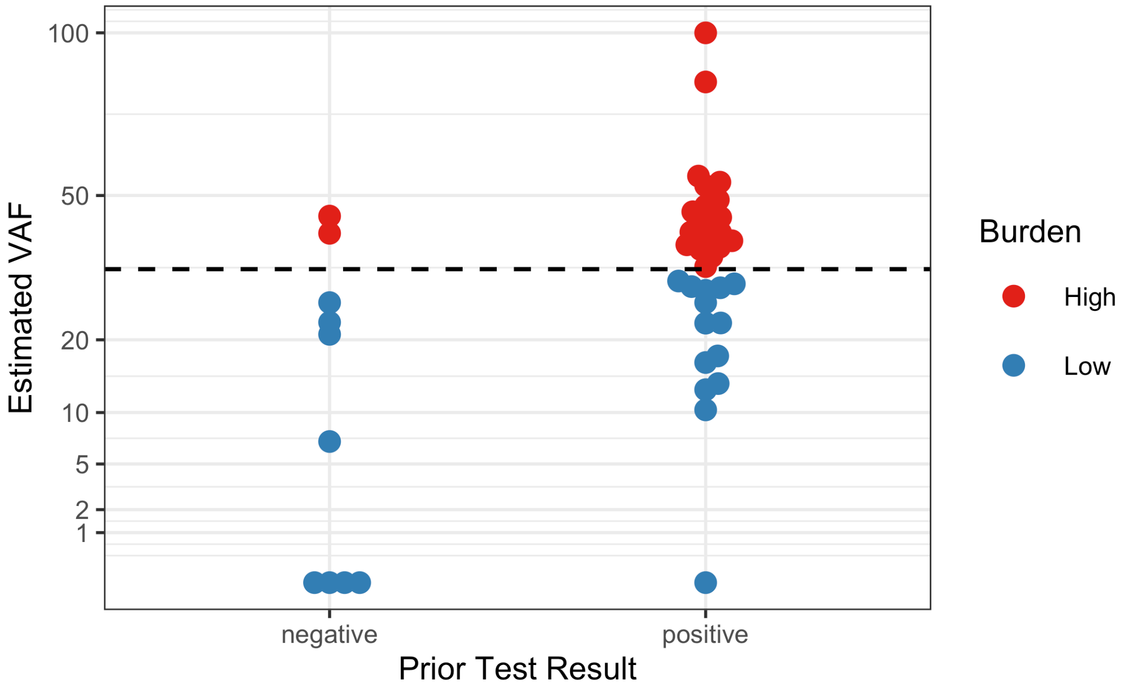

Supplementary Figure 9. Estimated VAF for *FLT3*-ITD events detected or not detected by prior clinical assay in the AML PMP exploratory cohorts. The horizontal dashed line indicates the estimated VAF of 33% used to discriminate high-burden from low-burden *FLT3*-ITD events.

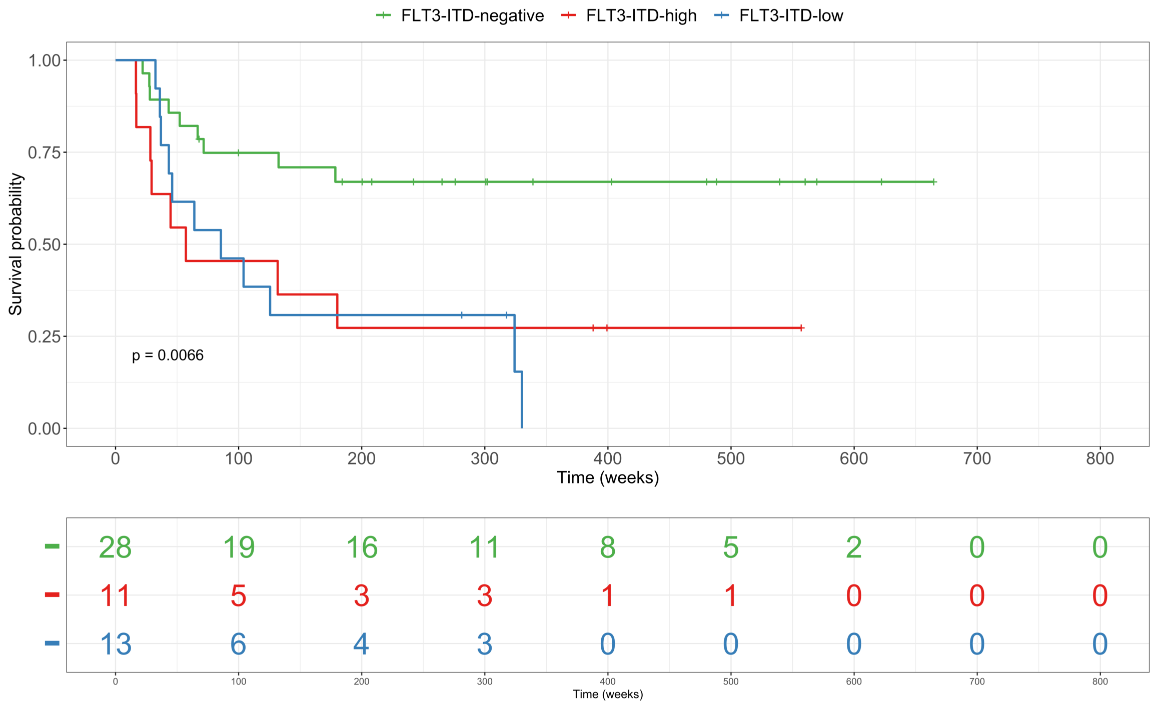

Supplementary Figure 10. Overall survival for normal-karyotype AML patients in the AML PMP exploratory cohorts by *FLT3*-ITD burden, compared to patients with no *FLT3*-ITD event.

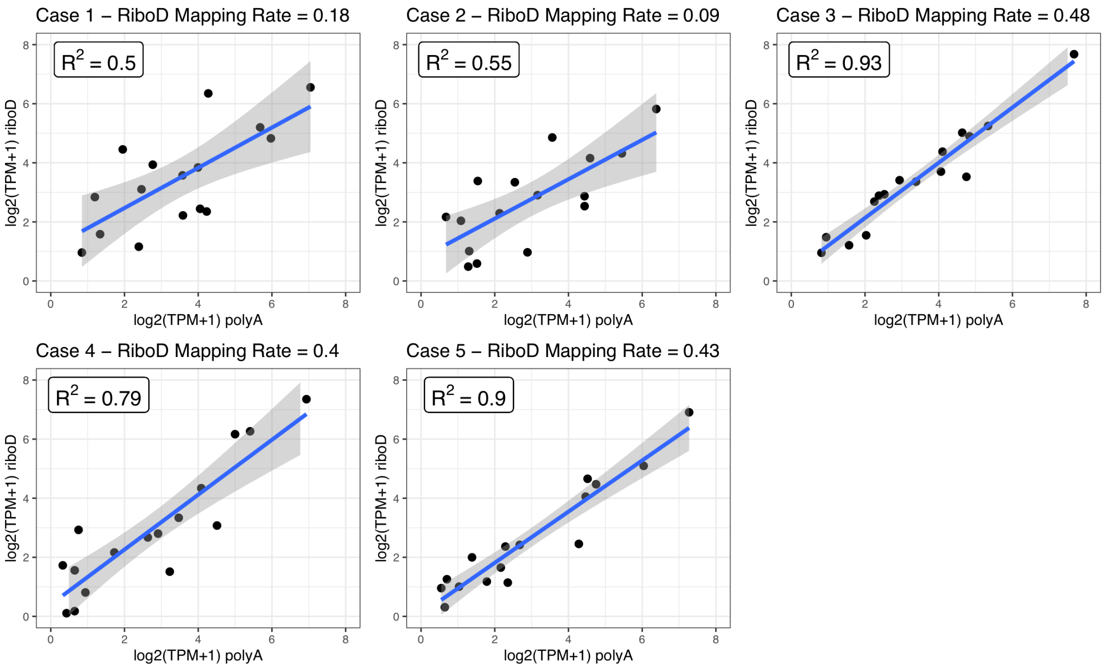

Supplementary Figure 11. Comparison between matched RNA-Seq libraries prepared with polyA or ribodepletion protocols. In each panel, the mapping rate for the ribodepleted library is indicated in the panel title. Each panel also indicates a linear regression of the two variables, with 95% confidence interval.

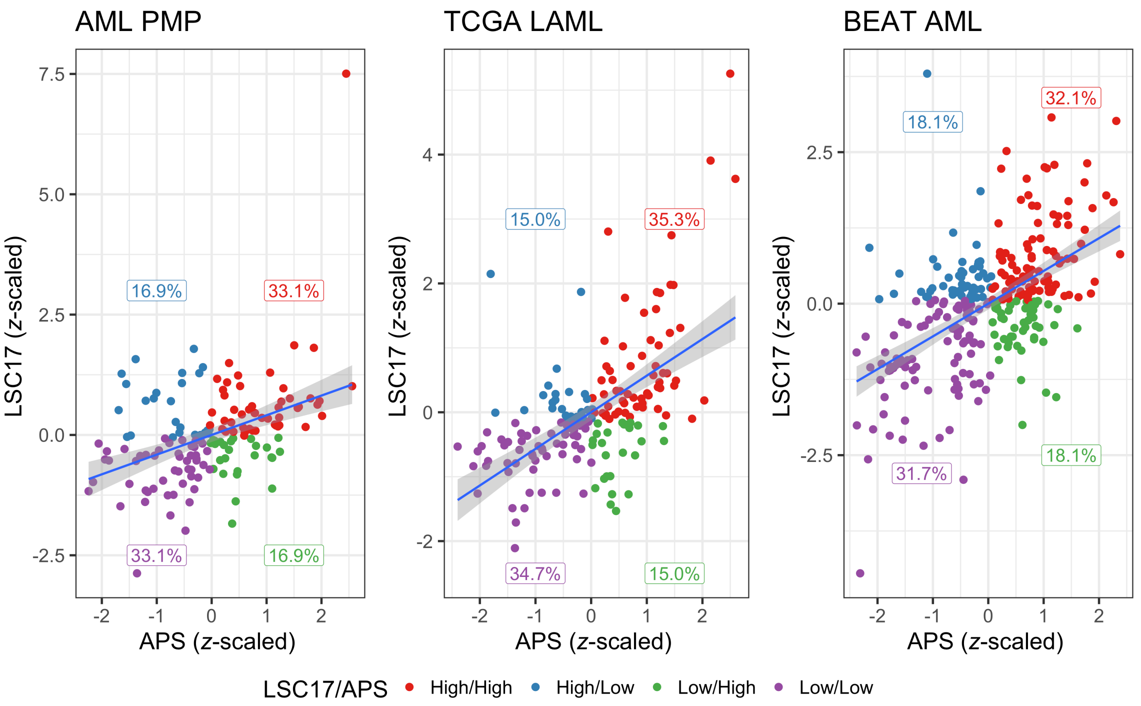

Supplementary Figure 12. Scaled APS and LSC17 values in the AML PMP, TCGA LAML, and BEAT AML cohorts. For each cohort, APS and LSC17 values were standardized by calculating *z* scores. High/low categories used for patient stratification are indicated by colour. Each panel also indicates a linear regression of the two variables (indicated with a blue line), with 95% confidence interval.

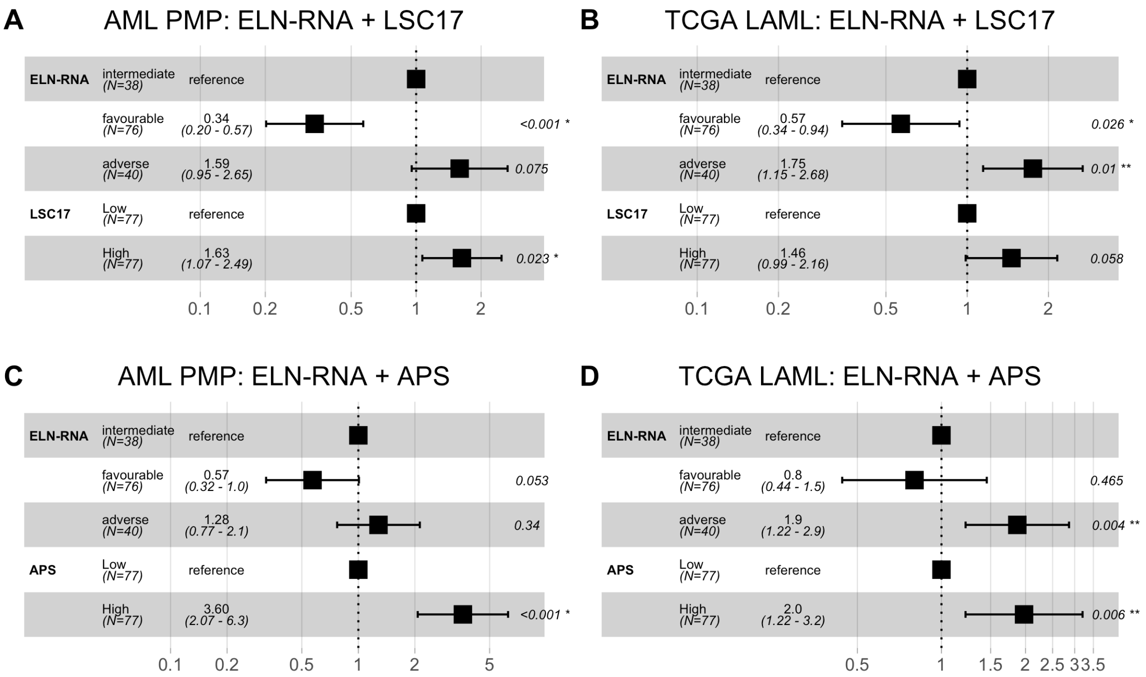

Supplementary Figure 13. Forest plots for multivariate survival models based on ELN-RNA stratifications with either the LSC17 or APS gene signatures. Multivariate Cox proportional hazards regression models were fit, modeling survival as a function of the ELN-RNA stratification and an expression score (either LSC17 or APS). In each panel, the hazard ratio is indicated on the x-axis, and model variables are indicated on the y-axis. In each model, 'Intermediate-risk' and 'Low' values for the gene signatures were set as the reference levels. In each panel, the estimated hazard ratio for a given variable is indicated by a square, with 95% confidence intervals. *p*-values associated with each variable are indicated at the right of each plot.

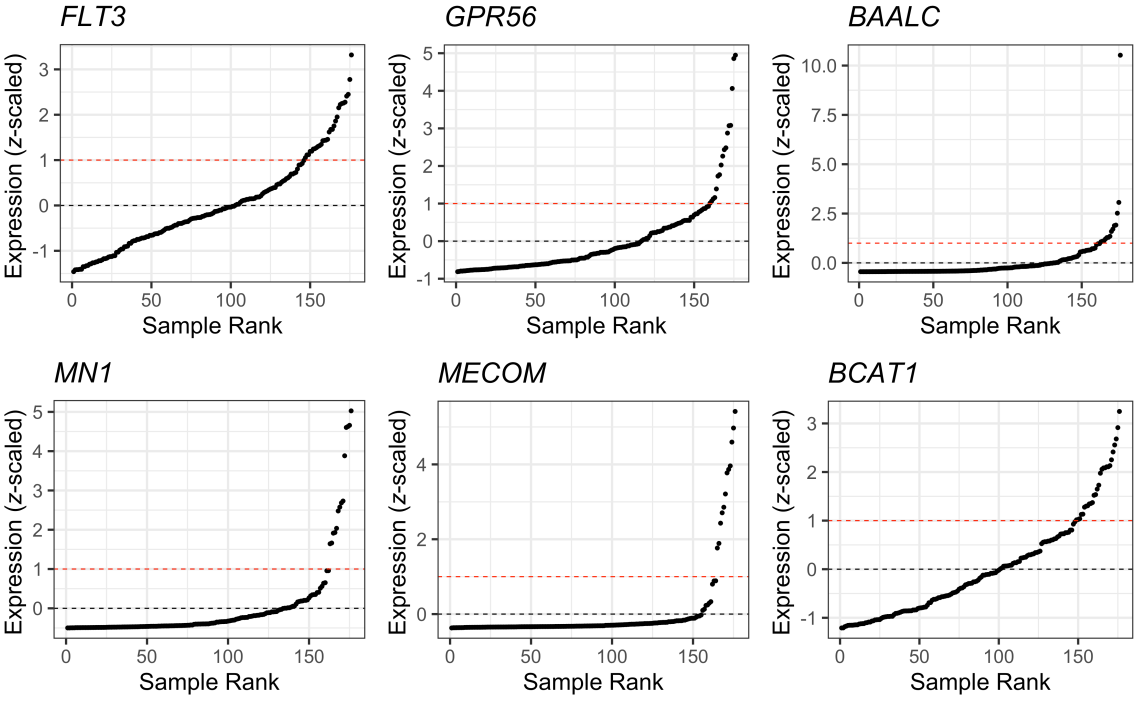

Supplementary Figure 14. Single-gene expression outliers. Samples are ranked by gene expression, measured as a z score (where $z = (x - \mu)/\sigma$). Black dashed line = mean expression, red dashed line = high outlier cutoff.

A

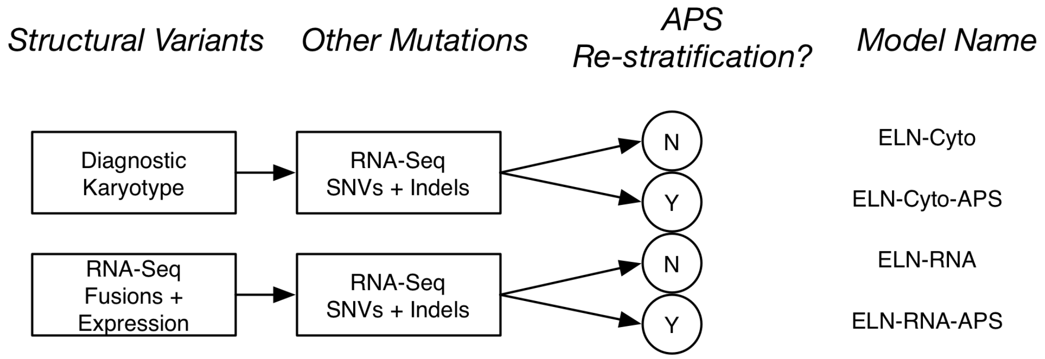

B

ELN-Cyto	ELN-RNA	n
Favourable	Favourable	74
Intermediate	Favourable	1
Intermediate	Intermediate	25
Intermediate	Adverse	7
Adverse	Favourable	1
Adverse	Intermediate	13
Adverse	Adverse	33
**Discrepant**		**22**

C

ELN-Cyto-APS	ELN-RNA-APS	n
Favourable	Favourable	77
Intermediate	Intermediate	6
Intermediate	Adverse	2
Adverse	Intermediate	7
Adverse	Adverse	62
**Discrepant**		**9**

Supplementary Figure 15. Comparison of patient stratification models. (**A**) Schematic overview of stratification models applied. (**B**) Comparison of patient stratifications between the ELN-Cyto and ELN-RNA models. (**C**) Comparison of stratifications between the ELN-Cyto-APS and ELN-RNA-APS models.

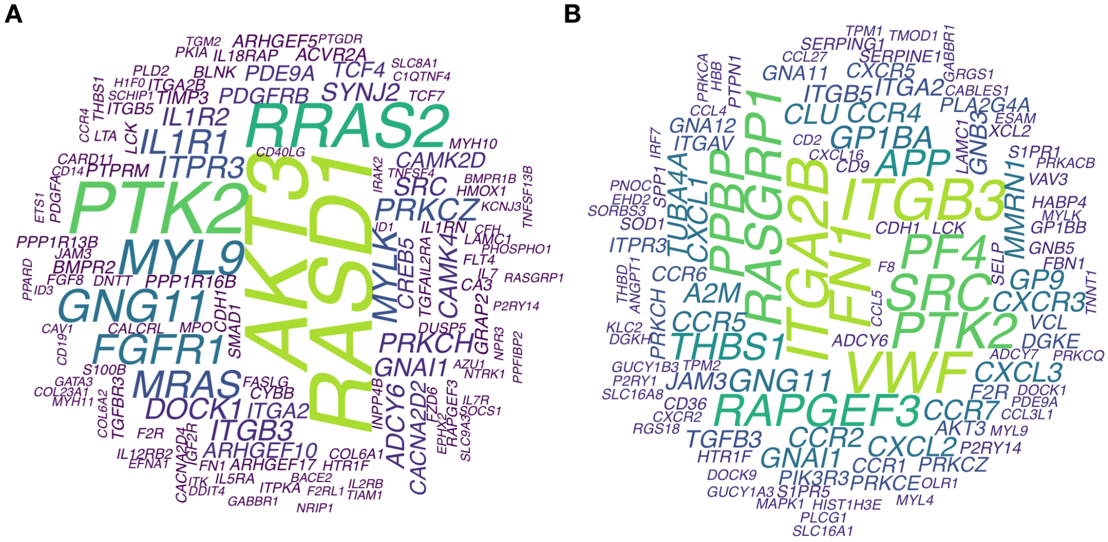

Supplementary Figure 16. Recurrently used molecules in enriched pathways from IPA (A) and GSEA (B) pathway enrichment analyses. For each analysis, IPA molecules or leading edge genes from enriched pathways were extracted and summarized, and visualized as word clouds.

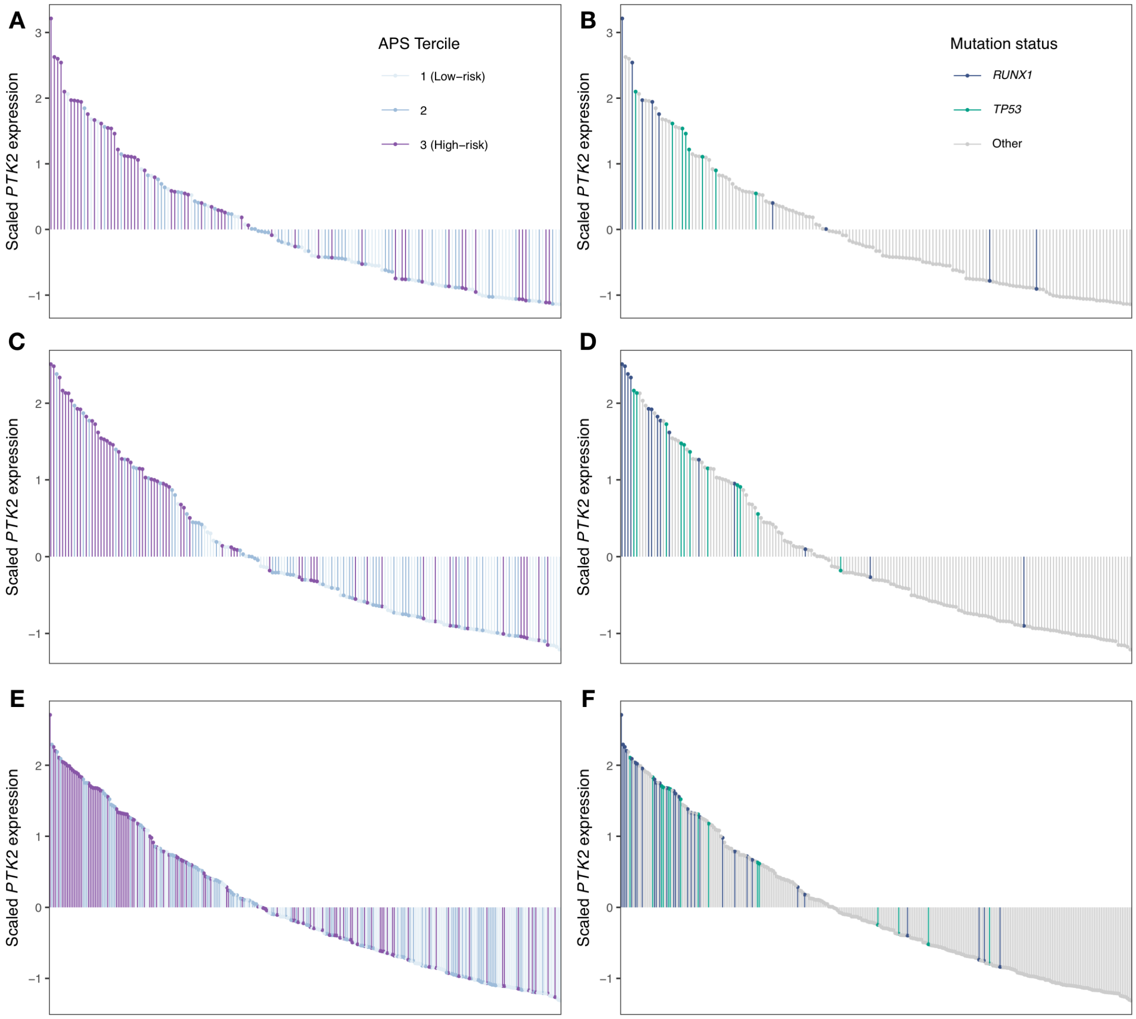

Supplementary Figure 17. Patients ranked by *PTK2* expression. (**A, C, E**) Patients are ranked by *PTK2* expression, and coloured by APS tercile for the AML PMP (**A**), TCGA LAML (**C**), and BEAT AML (**E**) cohorts. (**B, D, F**) Patients are ranked by *PTK2* expression, and coloured mutation status for the AML PMP (**B**), TCGA LAML (**D**), and BEAT AML (**F**) cohorts.

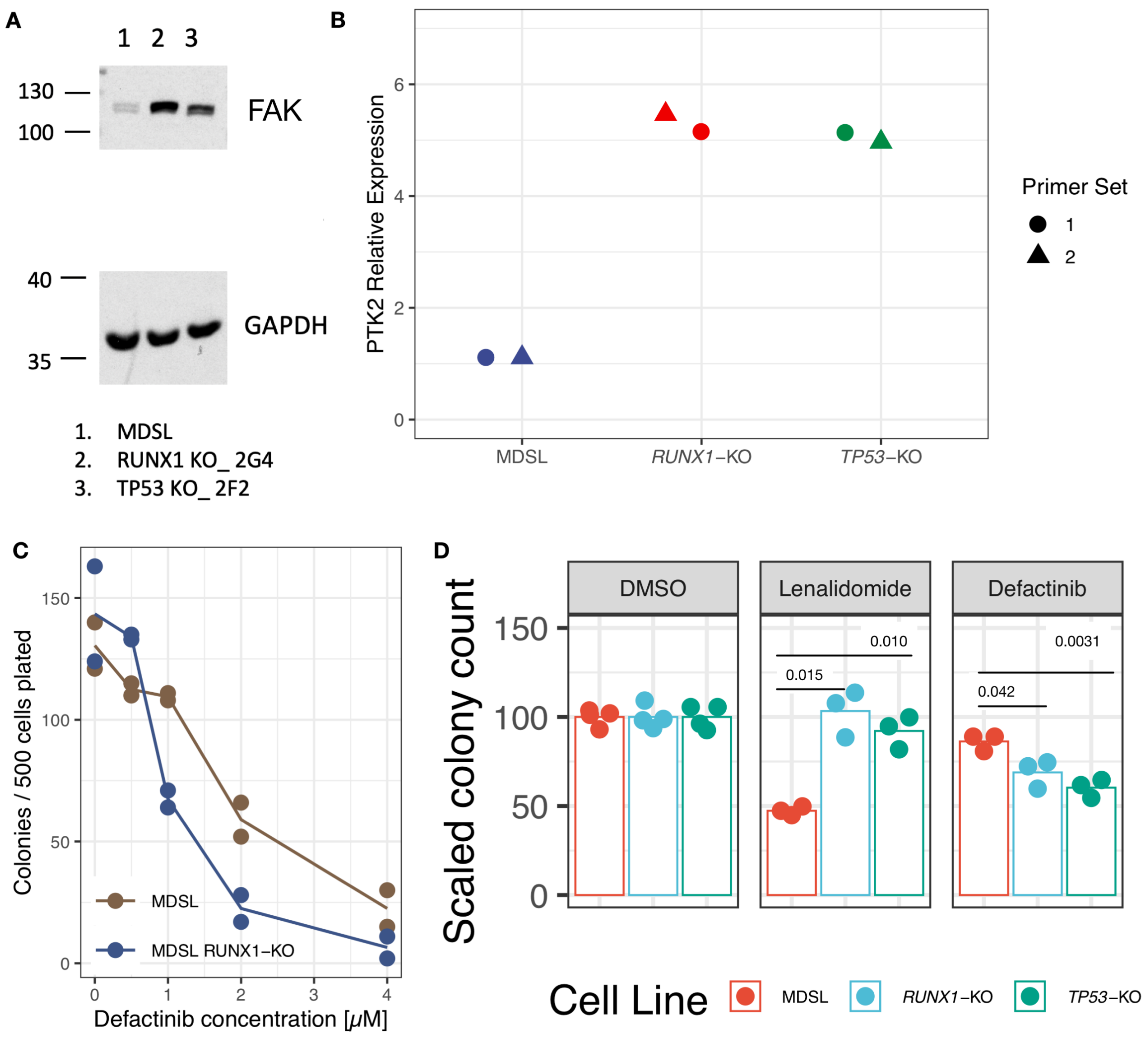

Supplementary Figure 18. FAK inhibition in MDSL cell line derivatives. (**A**) Western blot for FAK protein expression in MDSL cell lines with *RUNX1* or *TP53* CRISPR knockout, using cell lines generated by Martinez-Hoyer *et al.*.[1] Each blot was repeated twice, with similar results. (**B**) qPCR relative expression for *PTK2*. (**C**) Colony forming cell-count dose-response curve for defactinib. (**D**) Colony forming cell count assays for cells treated with DMSO, 1µm Lenalidomide, or 1µm Defactinib, with two-sided *t*-test *p* values indicated.

Supplementary References

1. Martinez-Høyer, S. *et al.* Loss of lenalidomide-induced megakaryocytic differentiation leads to therapy resistance in del(5q) myelodysplastic syndrome. *Nature cell biology* (2020). doi:10.1038/s41556-020-0497-9
