# Supplementary material for: A clinical transcriptome approach to patient stratification and therapy selection in acute myeloid leukemia
Source: Nat Commun. 2021 Apr 30;12:2474. doi: 10.1038/s41467-021-22625-y (PMC8087683; doi:10.1038/s41467-021-22625-y)
Supplement: Supplementary file 3 — Description of Additional Supplementary Files [file 41467_2021_22625_MOESM3_ESM.pdf]

Supplementary Data Legends

Supplementary Data 1. Cohort comparison for the AML PMP exploratory, TCGA LAML, and BEAT AML patient cohorts. Numbers within brackets represent the percentage within each cohort and category. 'ELN 2017' refers to patient stratifications by ELN criteria,[1] which are not applicable to MDS patients.

Supplementary Data 2. Sequencing libraries retained for analysis from the AML PMP cohorts.

Supplementary Data 3. Sample details and clinical characteristics for patients in the AML PMP exploratory cohort with AML, sAML, or tAML disease.

Supplementary Data 4. Sample details and clinical characteristics for patients in the AML PMP exploratory cohort with MDS or tMDS disease.

Supplementary Data 5. Sample details and clinical characteristics for patients and cell lines in the AML PMP validation cohort.

Supplementary Data 6. Sample details and clinical characteristics for patients in the AML PMP prospective cohort.

Supplementary Data 7. Sample details, clinical characteristics, and derived features for patients in the TCGA LAML cohort.[2]

Supplementary Data 8. Sample details, clinical characteristics, and derived features for patients in the BEAT AML cohort.[3]

Supplementary Data 9. Genes assessed for SNVs and short indels. Unless otherwised indicated in the 'Targets Assessed' column, all coding exons of selected transcripts were assessed.

Supplementary Data 10. Coverage summary statistics for observed SNV and short indel variants by sequencing platform and cohort. The 'Count' column indicates the total number of variants observed, while the remaining columns summarize the observed sequencing coverage depth at those sites.

Supplementary Data 11. Variants discordant between paired WGS and RNA-Seq libraries in the AML PMP exploratory batches.

Supplementary Data 12. Variants discordant between paired WES and RNA-Seq libraries in the AML PMP exploratory batches.

Supplementary Data 13. Variant annotation for variants not recovered by RNA-Seq in the AML PMP exploratory batches.

Supplementary Data 14. AML PMP validation cohort RNA-Seq samples. For each sample, the variants of clinical interest and number of replicate samples are indicated. Note that for samples 157-18 and 213-51, two of three replicates failed sequencing or sequencing quality checks, therefore these samples were excluded from further analysis.

Supplementary Data 15. Raw variant categories across variant callers in the validation cohort. Calls are summed across all replicate samples for each category and variant caller combination. 'FLT3-ITD' calls are additional SNV/indel calls neighbouring true *FLT3*-ITD events. 'Low coverage - FP', 'Low coverage - FN', and 'Low coverage - TP' sites

are FP, FN, and TP sites, respectively, with high-quality coverage depth < 10. ‘Known spurious’ sites are those corresponding to eight recurrent spurious variants.

Supplementary Data 16. Analytic validation of SNV calling for replicate cell line and patient material. For each of the three variant callers and the ensemble caller, SNV and short indel calls were compared to ground truth sets. The numbers of events were averaged across replicates for each cell line or patient sample. TP, true positive; FN, false negative; FP, false positive; Sensitivity = TP / (TP + FN); PPV, positive predictive value = TP / (TP + FP). Sensitivity and PPV are presented with 95% confidence intervals calculated by the binomial test.

Supplementary Data 17. Observed false-negative variants in the RNA-Seq validation cohort. Each row represents a single variant, with statistics for mean depth and VAF calculated from observations for different caller and replicate samples.

Supplementary Data 18. Observed false-positive variants in the RNA-Seq validation cohort. Each row represents a single GATK HaplotypeCaller variant variant call, with the observed depth and VAF.

Supplementary Data 19. Additional detected fusions with corresponding karyotype annotations in the AML PMP exploratory cohorts.

Supplementary Data 20. Recurrent artefactual and common fusion events in the AML PMP exploratory cohorts. ‘Recurrent Artefact’ and ‘Recurrent Common’ events were determined based on manual review of recurrence within the cohort, fusion properties, and literature review.

Supplementary Data 21. Detected fusions in AML-related genes in the AML PMP exploratory cohorts. For each novel fusion in an AML-related gene, we manually reviewed the RNA-Seq data in IGV. Additionally, where additional array, gene panel, WGS, or WES data was available, we reviewed those data sources.

Supplementary Data 22. Additional detected fusions in the AML PMP exploratory cohorts.

Supplementary Data 23. AML PMP exploratory patient cases with high *MECOM* and low *GATA2* expression.

Supplementary Data 24. Outlier *MECOM* expression in the TCGA LAML cohort. *MECOM* and *GATA2* expression values are *z*-scaled.

Supplementary Data 25. Model coefficients for the APS gene expression signature.

Supplementary Data 26. APS and LSC17 categories in the AML PMP, TCGA LAML, and BEAT AML cohorts. Each patient was classified as 'High' or 'Low' for the APS and LSC17 gene expression signatures as described in the text.

Supplementary Data 27. Univariate survival models, AML PMP exploratory AML samples (n = 154). Cox proportional hazard models were run for each indicated variable. For variables with discrete categories, the number of matching samples is indicated (n). The estimated hazard ratio for each variable is given in raw (HR) and log10-scaled units (log10(HR)). The 'Adj. p' column gives the FDR-adjusted *p* value, and the '-10*log10(Adj. p)' column gives the scaled, FDR-adjusted *p* value.

Supplementary Data 28. Modifications to ELN 2017 molecular guidelines used for patient stratification models. The ‘Cytogenetic Event’ column contains events inferred from karyotyping assays, while the ‘RNA-Seq Event’ column represents the same (or proxy) events inferred from the RNA-Seq assay. Events marked with (*) are modifications to the ELN2017 schema from Döhner et al.[1] For both stratification schemes, Cytogenetic and RNA-Seq Events take precedence over other mutational events.

Supplementary References

1. Döhner, H. *et al.* Diagnosis and management of AML in adults: 2017 ELN recommendations from an international expert panel. *Blood* **129,** 424–447 (2017).

2. Cancer Genome Atlas Research Network *et al.* Genomic and epigenomic landscapes of adult de novo acute myeloid leukemia. *The New England journal of medicine* **368,** 2059–2074 (2013).

3. Tyner, J. W. *et al.* Functional genomic landscape of acute myeloid leukaemia. *Nature* **562,** 526–531 (2018).
