# Supplementary material for: A clinical transcriptome approach to patient stratification and therapy selection in acute myeloid leukemia
Source: Nat Commun. 2021 Apr 30;12:2474. doi: 10.1038/s41467-021-22625-y (PMC8087683; doi:10.1038/s41467-021-22625-y)
Supplement: Supplementary file 5 — Reporting Summary [file 41467_2021_22625_MOESM5_ESM.pdf]

nature research

Corresponding author(s): Dr. Aly Karsan

Last updated by author(s): Jan 10, 2021

Reporting Summary

Nature Research wishes to improve the reproducibility of the work that we publish. This form provides structure for consistency and transparency in reporting. For further information on Nature Research policies, see our [Editorial Policies] and the [Editorial Policy Checklist].

Statistics

For all statistical analyses, confirm that the following items are present in the figure legend, table legend, main text, or Methods section.

n/a	Confirmed	
☐	☒	The exact sample size (*n*) for each experimental group/condition, given as a discrete number and unit of measurement
☐	☒	A statement on whether measurements were taken from distinct samples or whether the same sample was measured repeatedly
☐	☒	The statistical test(s) used AND whether they are one- or two-sided<br>*Only common tests should be described solely by name; describe more complex techniques in the Methods section.*
☐	☒	A description of all covariates tested
☐	☒	A description of any assumptions or corrections, such as tests of normality and adjustment for multiple comparisons
☐	☒	A full description of the statistical parameters including central tendency (e.g. means) or other basic estimates (e.g. regression coefficient) AND variation (e.g. standard deviation) or associated estimates of uncertainty (e.g. confidence intervals)
☐	☒	For null hypothesis testing, the test statistic (e.g. *F*, *t*, *r*) with confidence intervals, effect sizes, degrees of freedom and *P* value noted<br>*Give P values as exact values whenever suitable.*
☒	☐	For Bayesian analysis, information on the choice of priors and Markov chain Monte Carlo settings
☒	☐	For hierarchical and complex designs, identification of the appropriate level for tests and full reporting of outcomes
☐	☒	Estimates of effect sizes (e.g. Cohen's *d*, Pearson's *r*), indicating how they were calculated

Our web collection on statistics for biologists contains articles on many of the points above.

Software and code

Policy information about availability of computer code

Data collection

All software tools used for data collection are described in the Methods. The bioinformatics analysis pipeline relied on software tools including:

- bcbio-nextgen (version 1.0.1)
- BWA (version 0.5.7, ‘aln’ and ‘sampe’ subcommands)
- GSNAP (versions 2013-10-28 and 2014-12-28)
- RNA-SeQC (version v1.1.8)
- VarScan (version 2.4.2)
- GATK HaplotypeCaller (version 3.7)
- FreeBayes (version 1.1.0)
- vt (version 0.57)
- snpEff (version 4.3g)
- gemini (version 0.20.0)
- Trans-ABySS (version 1.5.2)
- PAVfinder (versions 0.2.0 and 0.3.0)
- BWA MEM (version 0.7.12-r1039)
- sailfish (version 0.9.0)
- preprocessCore (version 1.48.0)
- glmnet (version 4.0-2)
- Ingenuity Pathway Analysis (December 2018 Release)
- GSEA (version 3.0)
- EnrichR (version 2.1)

- DESeq2 (version 1.26.0)

Citations for these tools are provided in the manuscript.

Data analysis	All software tools used for data analysis are described in the Methods. Custom analysis tools developed for the manuscript are available at [https://doi.org/10.5281/zenodo.4411968].

For manuscripts utilizing custom algorithms or software that are central to the research but not yet described in published literature, software must be made available to editors and reviewers. We strongly encourage code deposition in a community repository (e.g. GitHub). See the Nature Research guidelines for submitting code & software for further information.

Data

Policy information about availability of data

All manuscripts must include a data availability statement. This statement should provide the following information, where applicable:

- Accession codes, unique identifiers, or web links for publicly available datasets
- A list of figures that have associated raw data
- A description of any restrictions on data availability

Sequence and clinical data associated with the AML PMP project is deposited at the European Genome-Phenome Archive under accession number EGAS00001004655. Raw data associated with all figures is available as Source Data.

The analysis also relied on database and annotation resources including:

- COSMIC (version 68)
- ExAC (r0.3)
- gnomAD (r2.0.1)
- ClinVar (v20160502)
- CADD (version 1.0)
- MSigDB hallmark gene set collection (v6.2)
- Reactome

Field-specific reporting

Please select the one below that is the best fit for your research. If you are not sure, read the appropriate sections before making your selection.

☒ Life sciences ☐ Behavioural & social sciences ☐ Ecological, evolutionary & environmental sciences

For a reference copy of the document with all sections, see nature.com/documents/nr-reporting-summary-flat.pdf

Life sciences study design

All studies must disclose on these points even when the disclosure is negative.

Sample size	The sample size of 176 patients in the retrospective dataset was selected so as to be comparable in size to other similar sequencing efforts (such as the TCGA LAML project (Cancer Genome Atlas Research Network, 2013)), which has previously been demonstrated to be sufficient for mutation and expression biomarker discovery.
Data exclusions	Some samples were excluded due to sample quality issues (described in the Methods). For some analyses, only AML-like patients were relevant to the question at hand, so MDS-like patients were excluded. For some differential expression analyses, acute promyelocytic leukemia (APL) patients were excluded (in order to consider expression differences between non-APL-like AML cases). Exclusion criteria were established through analysis of the retrospective analysis cohorts, then applied directly to the Validation cohorts.
Replication	To demonstrate the reproducibility of the informatics pipeline and library preparation methods, we prepared multiple cell line and patient libraries in triplicate. As described in the text, while most attempts at replication were successful, several replicates failed to meet the sample quality thresholds described above, and so were excluded.
Randomization	The samples in this study were pre-treated, so randomization into different experimental groups is not applicable. To control for disease-related covariates, the initial sample selection was constructed so as to include a broad representation of AML subtypes. Because of this, the distribution of AML subtypes in the study cohorts is not the same as the distribution in unselected patients.
Blinding	Blinding is not relevant to this study - the samples in this study were pre-treated, and the intent was to discover biomarkers using exploratory analysis methods.

Reporting for specific materials, systems and methods

We require information from authors about some types of materials, experimental systems and methods used in many studies. Here, indicate whether each material, system or method listed is relevant to your study. If you are not sure if a list item applies to your research, read the appropriate section before selecting a response.

Materials & experimental systems

n/a	Involved in the study	
[ ]	[x]	Antibodies
[ ]	[x]	Eukaryotic cell lines
[x]	[ ]	Palaeontology and archaeology
[x]	[ ]	Animals and other organisms
[ ]	[x]	Human research participants
[x]	[ ]	Clinical data
[x]	[ ]	Dual use research of concern

Methods

n/a	Involved in the study	
[x]	[ ]	ChIP-seq
[x]	[ ]	Flow cytometry
[x]	[ ]	MRI-based neuroimaging

Antibodies

Antibodies used: anti-RUNX1 (CST 4334, 1:1000), anti-PTK2 (CST 13009, 1:1000), anti-GAPDH (CST 2118, 1:5000)

Validation:
anti-RUNX1: https://www.cellsignal.com/products/primary-antibodies/aml1-antibody/4334
anti-FAK: https://www.cellsignal.com/products/primary-antibodies/fak-d2r2e-rabbit-mab/13009
anti-GAPDH: https://www.cellsignal.com/products/primary-antibodies/gapdh-14c10-rabbit-mab/2118

Eukaryotic cell lines

Policy information about cell lines

Cell line source(s): Cell lines used in the study include:

- NA12878 (Coriell)
- NA19240 (Coriell)
- HMC-1
- KASUMI-6 (DSMZ)
- MOLM-13 (DSMZ)
- OCI-AML3 (DSMZ)
- MDSL
- KG1a (ATCC)
- THP-1 (ATCC)
- UT-7 (DSMZ)

Authentication: Cell lines were validated using STR profiles against known profiles.

Mycoplasma contamination: All cell lines tested negative for mycoplasma contamination.

Commonly misidentified lines (See ICLAC register): Of the cell lines used in this study, only UT-7 is listed in the ICLAC, which indicates that the correct version is held at DSMZ (where we obtained the cell line).

Human research participants

Policy information about studies involving human research participants

Population characteristics: Population characteristics are described in Table S1. For each of the experimental cohorts, complete annotations are included as Source Data. The covariate-relevent population characteristics of the human research participants (the AML PMP retrospective AML cases) include: Age (120 <60 years, 34 >= 60 years), Sex (78 Female, 76 Male), ELN 2017 Risk Status (74 favourable, 33 intermediate, 47 adverse).

Recruitment: For the AML PMP dataset, patients were selected from existing available material at our research institute - no patients were recruited specifically for this study.

Ethics oversight: Ethics protocols were approved by the BC Cancer Research Ethics Board, under protocols H04-61292, H09-01779, H11-01484, and H13-02687

Note that full information on the approval of the study protocol must also be provided in the manuscript.
